# Automating high-throughput screening for anthracnose resistance in common bean using allele specific PCR

**DOI:** 10.1186/s13007-023-01071-5

**Published:** 2023-10-03

**Authors:** Marysia Zaleski-Cox, Phillip N. Miklas, Alvaro Soler-Garzón, Valerio Hoyos-Villegas

**Affiliations:** 1 Department of Plant Science, McGill University, Montreal, QC Canada; 2 Grain Legume Genetics and Physiology Research Unit, USDA-ARS, Prosser, WA USA; 3 Irrigated Agriculture Research and Extension Center, Washington State University, Prosser, WA USA

**Keywords:** Anthracnose, KASP assay, High-throughput genotyping, Lab automation, Marker-assisted selection, *Phaseolus vulgaris*

## Abstract

**Background:**

Common beans (*Phaseolus vulgaris* L.) provide important protein and calories globally. Anthracnose (*Colletotrichum lindemuthianum* (Sacc. & Magnus) Briosi & Cavara, 1889) is a major disease in common bean and causes significant yield losses in bean production areas. Screening for markers linked to known disease resistance genes provides useful information for plant breeders to develop improved common bean varieties. The Kompetitive Allele Specific PCR (KASP) assay is an affordable genetic screening technique that can be used to accelerate breeding programs, but manual DNA extraction and KASP assay preparation are time-consuming. Several KASP markers have been developed for genes involved in resistance to bean anthracnose, which can reduce yield by up to 100%, but their usefulness is hindered by the labor required to screen a significant number of bean lines. Our research objective was to develop publicly available protocols for DNA extraction and KASP assaying using a liquid handling robot (LHR) which would facilitate high-throughput genetic screening with less active human time required. Anthracnose resistance markers were used to compare manual and automated results.

**Results:**

The 12 bean anthracnose differential cultivars were screened for four anthracnose KASP markers linked to the resistance genes *Co-1*, *Co-3* and *Co-4*^*2*^ both by hand and with the use of an LHR. A protocol was written for DNA extraction and KASP assay thermocycling to implement the LHR. The LHR protocol reduced the active human screening time of 24 samples from 3h44 to 1h23. KASP calls were consistent across replicates but not always accurate for their known linked resistance genes, suggesting more specific markers still need to be developed. Using an LHR, information from KASP assays can be accumulated with little active human time.

**Conclusion:**

Results suggest that LHRs can be used to expedite time-consuming and tedious lab work such as DNA extraction or PCR plate filling. Notably, LHRs can be used to prepare KASP assays for large sample sizes, facilitating higher throughput use of genetic marker screening tools.

**Supplementary Information:**

The online version contains supplementary material available at 10.1186/s13007-023-01071-5.

## Background

Common beans (*Phaseolus vulgaris* L.), such as dry, green, or string beans, are an important source of calories, fiber and protein globally [1]. They contain 25% protein and are an affordable, healthy protein source with low environmental impact [2, 3]. There are two main genetic pools of bean diversity: Andean, including kidney beans or cranberry beans, and Middle American, including black or navy beans [4]. Global demand for pulses is increasing, so improvement efforts must accelerate to facilitate yield increases [5].

Many widespread bean diseases in North America have the potential to significantly reduce yield [6]. Resistance genes for prevalent diseases, such as anthracnose, must be identified and introgressed into lines widely grown in Quebec to combat yield loss. Presently, the average bean yield in Quebec is 2.3 t/ha but breeding improved varieties could enable growers to surpass 2.7 t/ha [7]. Improving disease resistance would increase yield, but it would also alleviate the financial burden on farmers caused by current disease management practices, such as purchasing clean seed every year and applying additional fungicide.

Anthracnose (*Colletotrichum lindemuthianum* (Sacc. & Magnus) Briosi & Cavara, 1889) is a seed-borne fungus that, if successful, can reduce yield by 100% [6]. Viable pods produced by an infected plant will contain infected seeds [8]. Severely infected seeds are often misshapen, but even seeds without visible symptoms will have anthracnose on their seed surface. Planting infected seeds can initiate anthracnose epidemics. Anthracnose exhibits significant physiological variability, with reports of more than 100 races worldwide. In Eastern Canada, race 73 predominates [9, 10], (Corkel and Hoyos-Villegas, unpublished observations). Races of anthracnose are identified by inoculating a set of 12 binary differential bean cultivars and observing their susceptibility to the applied fungus [11].

Over 20 genes conditioning anthracnose resistance in beans have been identified, a number which has been increasing over the past two decades [12]. The identified genes include *Co-1* to *Co-17* as well as *Co-u, Co-v, Co-w, Co-x, Co-y, Co-*z and others [13–33]*.* These genes span seven main chromosomal regions: Pv01, Pv02, Pv03, Pv04, Pv07, Pv08, and Pv11 [12]. The most important resistance genes are *Co-1* to *Co-5*, several of which are multi-allelic [8]. It is thought that anthracnose follows the gene-for-gene model, so each gene in the host confers resistance to specific anthracnose races [34]. 

KASP assays can be applied in marker-assisted selection (MAS) disease resistance breeding programs to contribute genetic gains by facilitating selections for desired genotypes [35]. KASP assays are a type of modified qPCR (quantitative PCR) that can genotype a codominant marker in a single step using allele-specific fluorescence. KASP is similar to standard PCR techniques except two forward primers are used, one for each allele version of a codominant marker such as a SNP or InDel. KASP forward primers contain different tail sequences that are complementary to cassettes with quenched fluorophores in the PCR master mix. Hexachloro-fluorescein (HEX) is associated with one allele tail, and Fluorescein amidites (FAM) is associated with the other. As the cycles of PCR progress, different amounts of the fluorescence connected to one or the other allele are accumulated. By quantifying the fluorescence at the end of thermocycling, the marker allele(s) present in a DNA sample can be determined [36]. In addition to being simple to use, KASP genotyping is also affordable. The cost of KASP was calculated to be less than half of the price TaqMan, another one-step genotyping technique [37]. Unfortunately, extracting DNA and preparing plates for thermocycling, required for a KASP assay, can be time-consuming if done manually. 

The objective of this research was to develop a publicly available, high-throughput protocol for DNA extraction and PCR assay preparation, using KASP assays as an example for validation. In addition, the predictability of four KASP markers for detecting associated anthracnose resistance genes was evaluated with assays of the anthracnose bean differentials.

Two hypotheses were tested concurrently. First, whether bean cultivars are assayed by hand or with the LHR, both methods will produce KASP assays with the same calls for the presence or absence of anthracnose resistance loci. Second, the presence or absence of fluorescence connected to the anthracnose resistance linked KASP marker assays will correctly identify known resistance loci present in the host differential cultivars.

## Results

### DNA quality

**Fig. 1:**
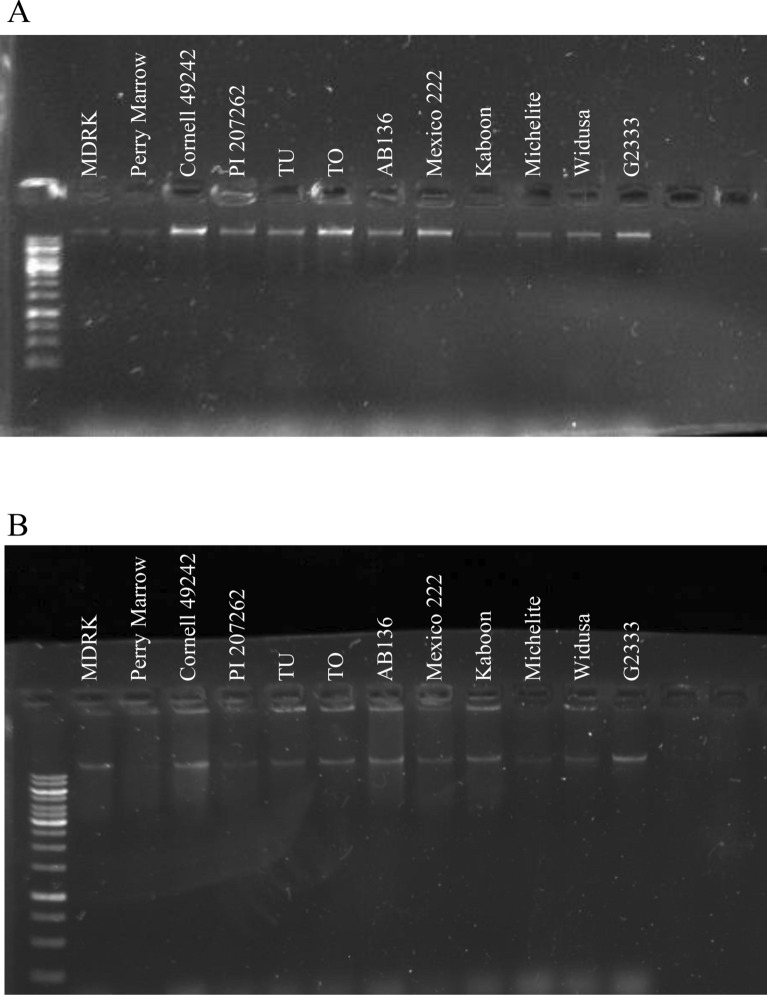
DNA extracted from the 12 anthracnose differential bean cultivars manually versus DNA extracted using an LHR. All samples were diluted to appropriate KASP concentrations. **A**: DNA extracted by the LHR using the MagMAX^™^ DNA extraction kit. All steps of extraction following lysis were performed by the LHR. **B**: DNA extracted manually using the MagMAX^™^ extraction kit

DNA extraction by hand or with the LHR provided sufficient quality DNA for KASP assays. DNA concentrations manually extracted from bean differential cultivar seed using the MagMAX^™^ extraction kit ranged from 503 to 1926 ng/μL with a mean concentration of 1038 ng/μL. DNA concentrations from bean differential cultivar seed using the same kit but implementing the LHR ranged from 23.8 to 662 ng/μL with a mean concentration of 291 ng/μL. All DNA extracted manually or with the LHR using either extraction kit (DNeasy^®^ Plant Pro Kit or MagMAX^™^ Plant DNA Isolation Kit) was sufficient for KASP assays (Fig. 1). DNA was diluted to a final concentration of 20–50 ng/μL for KASP assays [36].

### Call accuracy

**Fig. 2:**
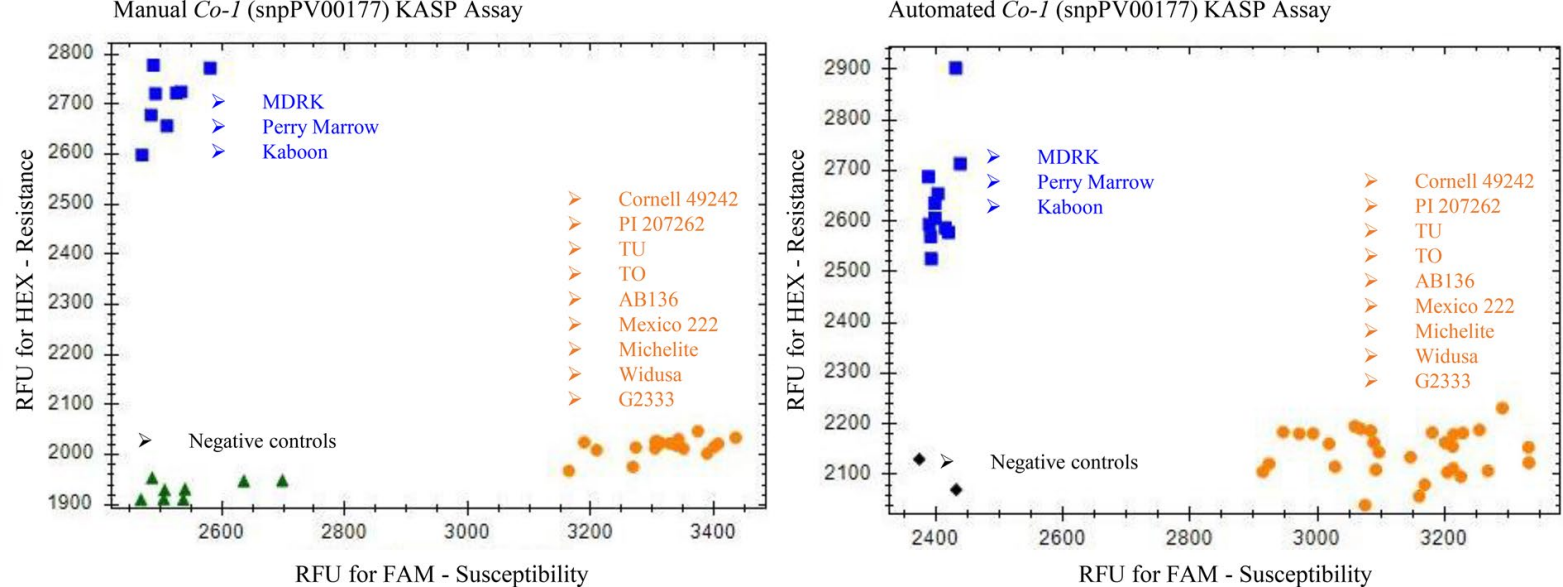
Comparison of KASP results for the *Co-1* marker (snpPV00177) when conducted manually or by the LHR on the anthracnose differential bean cultivars. Cultivars that are homozygous for resistance at the *Co-1* marker appear blue, while homozygous susceptible cultivars appear orange. No template controls are either green or black. Two technical replicates are shown for the differential cultivars as well as for a homozygous resistance and susceptible check line in the manual results. Three technical replicates for the differentials and check lines are shown for the automated assay, which accounts for the larger number of sample points on the automated graph

Presence or absence of the resistance gene marker was determined for the differential anthracnose cultivars and several check lines. These marker results were partially as expected according to present knowledge concerning anthracnose resistance loci. For the *Co-1* marker, snpPV00177 on Pv01 (Table 2), 11/12 calls were as expected on the anthracnose differential cultivars for this marker (Table 1). In addition, all *Co-1* marker calls made using an LHR matched those of the assay performed manually (Fig. 2). Manual and automated KASP assay plots from the three additional markers (snpPV00050, snpPV00070 and snpPV00183) can be seen in Additional file 1: Figure S1.

**Table 1:**
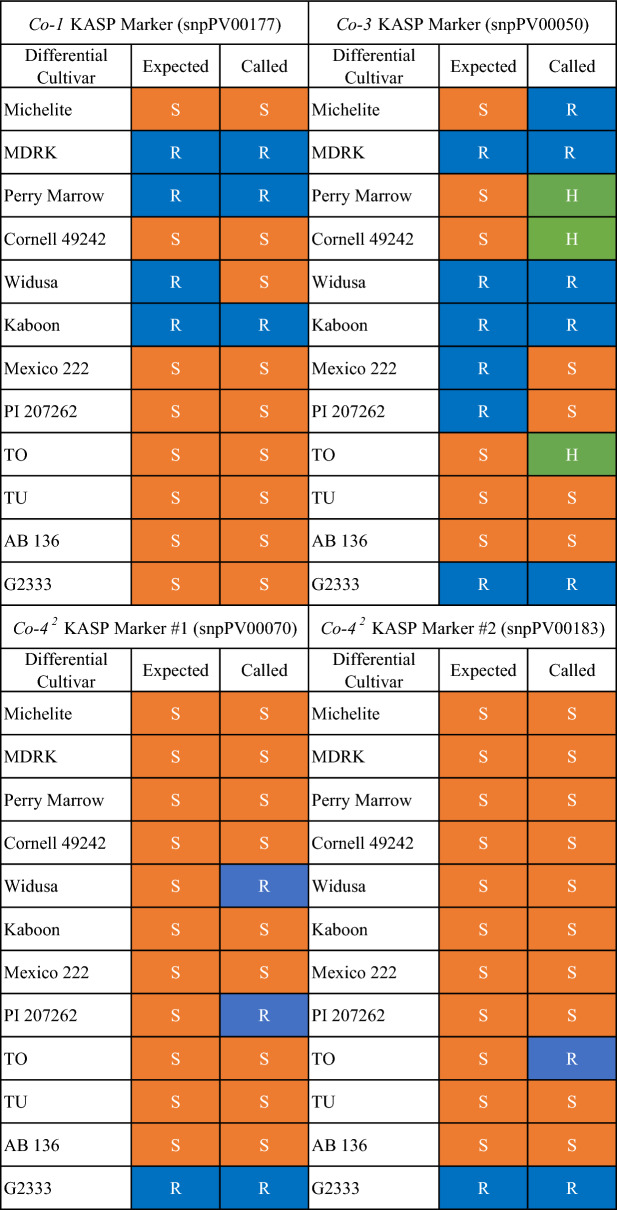
Expected versus actual calls for the tested KASP markers

Predicted KASP assay results at markers linked to anthracnose resistance genes based on known resistance loci in the anthracnose differential bean cultivars [8, 59] and observed results. S (orange) = susceptible, R (blue) = resistant, H (green) = heterozygous.

### Automation’s influence on results

The LHR was able to perform a KASP assay with the same calls as when executed manually 94% of the time. On rare occasions, the calls mismatched between the methods (3.8%), or no call was made by one method (1.9%). For three of the four markers, the same calls were made 100% of the time. All errors were connected to one marker, linked to *Co-3* (snpPV00050).

### Time saved

**Fig. 3:**
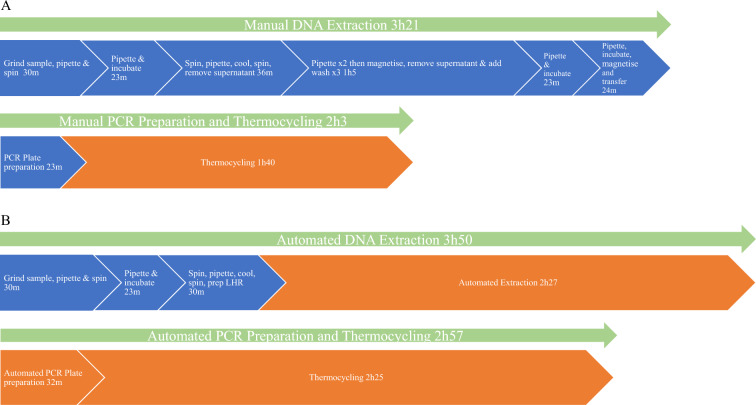
Time required for KASP assaying of 24 samples. Blue represents active human time required while orange represents automated time. **A** Manual KASP assay **B** Automated KASP assay

The active human time required to assay 24 samples from seed tissue to KASP plot decreased from 3h44 to 1h23 with the help the LHR. These figures include 30 minutes of manual sample preparation time. Total time increased somewhat when using the LHR from 5h24 when done manually to 6h47, mostly because of a slower thermocycler in the LHR which took 45 min longer to perform the same cycling procedure (Fig. 3).

## Discussion

### Repeatability versus specificity

The KASP markers proved to be highly repeatable. Across replicates and methods, the same calls were made 94.3% of the time. However, the highly repeatable calls were occasionally contradictory to what was expected given the resistance loci known to be present in the anthracnose differential cultivars. Markers had low to moderate specificity to their linked locus of interest suggesting the tested KASP markers are fit for identifying certain but not all allelic versions of some anthracnose resistance genes. Perhaps these markers are too far from their linked locus of interest to be specific to their targeted resistance gene. This issue could be resolved by developing markers more tightly linked to loci of interest. The expected versus observed results for each marker are listed in Table 1 and discussed below. Calls were less tightly clustered when the assay was performed by the LHR. The LHR used cannot execute a ramp of less than 1 °C per cycle, but a 0.8 °C ramp is optimal for KASP assays, which could explain the looser clusters in automated assays (Fig. 2, Additional file 1: Figure S1).

### Low cost and high-throughput

This work suggests an Opentrons OT-2 LHR is an affordable and effective tool for KASP assaying. Including all the modules required for extraction and thermocycling, the OT-2 costs $26 470 USD. Although only four markers were tested, the protocol provided does not change depending on the marker, so we expect it to be effective for DNA extraction and KASP assay amplification at any KASP marker. Additionally, many open-source protocols are publicly available on the Opentrons website. This protocol gives labs the ability to apply high volumes of automated KASP assays. Low initial costs and open-source protocols increase the accessibility of high throughput marker-assisted selection in breeding labs.

### Less active human time

Using the LHR, the time required to assay 24 samples (two biological replicates of the differential anthracnose cultivars) with KASP was reduced from 3h44 of active human labor to 1h23 (Fig. 3), including the time it takes to manually prepare and grind seed samples. This step would be much quicker if using fresh tissue samples. A test of 24 samples was chosen because it represents the most biological replicates of the anthracnose differential cultivars that can be loaded on to a 96 well PCR plate with three technical replicates per sample as well as adequate checklines and controls. The LHR still takes several hours to complete extractions and thermocycling, but it does not require human attention, freeing up time for other lab work and ultimately saving money.

### Discrepancies between *Co-1* KASP marker and known genotypes

The results of the *Co-1* KASP assay were mostly as expected, with Michigan Dark Red Kidney (MDRK), Perry Marrow, and Kaboon appearing as homozygous resistant at the *Co-1* marker (snpPV00177) while all the other differentials were called as homozygous susceptible. Oddly, Widusa was not called as possessing the allele linked to anthracnose resistance from *Co-1* despite possessing *Co-1*^*5*^, an allelic form of the *Co-1* gene as reported by [17]. The same result was found in the original validation of an InDel marker (NDSU_IND_1_50.2219) associated to the *Co-1* locus in the same study which discovered the snpPV00177 marker [12]. Marker based screening for resistance in Widusa using NDSU_IND_1_50.2219 predicted a homozygous susceptible response, as observed with the snpPV00177 marker, despite a phenotypic reaction of resistance when screened against race 73 of anthracnose [12]. Finer mapping of the *Co-1* genomic region for Widusa may elucidate this unexpected result. Ideally, markers specific to each of the allelic forms of *Co-1* would be found, enabling differentiation between allelic versions of the *Co-1* gene.

### Discrepancies between the *Co-3* KASP marker and known genotypes.

When testing the *Co-3* marker (snpPV00050), the anthracnose differentials Mexico 222 and PI 207262 were false negatives, as the KASP assay should have indicated that they possess the allele linked to anthracnose resistance at *Co-3* but did not. When tested, Widusa possessed the resistance allele of the *Co-3* marker, perhaps because Widusa contains an allelic form of the *Co-9* gene, which is clustered with the *Co-3* gene on Pv04 [8, 38]. It is unclear why Michelite was called as resistant. These results suggests that the exact specificity of the *Co-3* marker must be determined before this marker can be useful.

### Discrepancies between the *Co-4*^*2*^ KASP markers and known genotypes

In the differentials test of the first *Co-4*^*2*^ marker (snpPV00070), cultivars Widusa, PI 207262, G2333, and USPT-ANT-1 were called as containing the version of the marker indicating the presence of *Co-4*^*2*^ resistance. Widusa contains no known *Co-4* genes and PI 207262 contains a different *Co-4* allele, *Co-4*^*3*^. Additionally, TO, possessing *Co-4*, was not called as resistant with this marker.

In the test of the second *Co-4*^*2*^ KASP marker (snpPV00183) different, but similarly confounding results were found. G2333, USPT-ANT-1 (checkline) and TO were called resistant by the assay, which demonstrates that this marker is not specific to exclusively the *Co-4*^*2*^ allelic form. While G2333 and USPT-ANT-1 are known to contain *Co-4*^*2*^, TO contains a different allelic form, *Co-4*, as mentioned previously. It is worth noting that this *Co-4*^*2*^ KASP assay (snpPV00183) called the anthracnose differential cultivar PI 207262, containing *Co-4*^*3*^, as susceptible suggesting that the marker is specific to several but not all allelic forms of *Co-4.* Being specific to some but not all allelic forms is a significant error because the different allelic forms confer resistance to unique combinations of anthracnose races [11]. As with the *Co-1* marker tested, markers specific to just one allelic form of a locus would be ideal. Together, the results of the two *Co-4*^*2*^ markers suggest that there is presently no known KASP marker specific to only *Co-4*^*2*^ that could be used reliably for genetic screening if there were other *Co-4* allelic forms in the population.

### Future research

KASP assays need additional research to further improve high throughput application. Broadly, more tightly linked, specific markers are needed to improve the usefulness of these assays. Even if the KASP assay functions correctly, its usefulness is compromised if the call is not reliably linked to a locus of interest. Additionally, new technologies, such as multiplexing KASP assay mastermixes, would further increase throughput of the assay. With a multiplexed assay, multiple markers connected to several different loci of interest could be assayed at once. At least one multiplexing mastermix is currently being sold that assays two SNPs at a time [39]. If the number of SNPs that can be assayed together is increased to four or five, screening costs could be significantly reduced. Multiplexing mastermixes that assay two SNPs at a time are currently available in the market [39]. If the number of SNPs that can be assayed together is increased to four or five, screening costs could be significantly reduced.

Improvements within the scope of this research are possible, including applying assays to early-generation breeding lines, validating marker calls with pathogen testing and quicker extraction techniques. KASP markers would be especially useful if we could select for them before populations reach inbred status. F2–F4 selection would be ideal, because heterozygous individuals and those homozygous for susceptibility-linked marker alleles, could be discarded right away. Fewer plants or lines advanced in subsequent generations would save time and space in breeding programs. Pathogen testing is cumbersome and imperfect because it depends on uniform inoculation and controlled environments to work; however, marker utility for selecting specific genes can only be conclusively confirmed by exposing beans to appropriate anthracnose races. Finally, there are many different and faster extraction techniques to try. Quality can be compromised when quicker techniques are used but given that extraction is by far the most time-consuming part of the assay, methods such as Whatman FTA paper extraction should be tested.

## Conclusions

Using an LHR, information from KASP assays can be accumulated with little active effort and low costs. KASP assays for the markers selected were highly repeatable but did not completely reflect what is known about anthracnose resistance loci in the anthracnose differential cultivars. Using the LHR, the active time required to assay samples dropped without changing results. These results suggest that KASP can easily be applied as an effective genetic screening tool to conduct marker-assisted selection quickly across many individuals.

## Methods

The aim of this work was to test an automated system for KASP assaying from raw samples to fluorescence results and to validate its efficacy. To do this, KASP assays of four KASP markers were done with manual extraction and pipetting, and then the same assays were repeated using an LHR as much as possible. The resulting PCR products of both techniques were compared to ensure automation did not compromise the assay quality. With a goal of implementing MAS for anthracnose resistance in our breeding program we choose markers linked with the *Co-1*, *Co-3*, and *Co-4*^*2*^ resistance genes (Table 2). The primer sequences can be seen in Additional file 2: Table S1. Genome wide association studies (GWAS) found the SNP markers ss715645251on Pv01 and ss715640025 on Pv04 to be associated with the *Co-1* and *Co-3* resistance loci, respectively [40]. SNP ss715645251 was strongly associated with resistance to anthracnose races 65, 73, and 3481 while SNP ss715640025 was associated with resistance to anthracnose race 109. The SNP markers ANT_Co-4_08_TG_2391836 and S08_2443578 on Pv08, linked with *Co-4*^2^, were based upon fine mapping results for the *Co-4* locus [41, 42]. The tested *Co-4*^*2*^ SNPs themselves were identified using with available whole genome sequencing [43]. The Intertek (London, UK) IDs of the selected markers are snpPV00177 (*Co-1*), snpPV00050 (*Co-3*), snpPV00070 (*Co-4*^*2*^) and snpPV00183 (*Co-4*^*2*^). More information about these markers [44] and their sequences can be found at [43, 45] (Table 2, Additional file 2: Table S1).

**Table 2:** Tested KASP marker information

SNP ID	Associated resistance gene	Differential cultivar with associated gene	Intertek ID	Chromosome	Position (G19833 v2.1)	Source
ss645251	*Co-1*	MDRK, Kaboon, Perry Marrow, Widusa	snpPV00177	1	49,583,965	[10, 19]
ANT_Co-3_ss715640025	*Co-3*	Mexico 222, PI 207262, MDRK, Kaboon, Widusa, G2333	snpPV00050	4	169,725	[10, 19]
ANT_Co-4_08_TG_2391836	*Co-4*^*2*^	G2333	snpPV00070	8	2,324,579	[20–22]
S08_2443578	*Co-4*^*2*^	G2333	snpPV00183	8	2,443,578	[20–22]

Four checklines were assayed in addition to the anthracnose differential cultivars: Montcalm (*Co-1* checkline), G122 (*Co-3* checkline), USPT-ANT-1 (*Co-4*^*2*^ checkline) and Othello (susceptible checkline). Montcalm is a dark red kidney variety [46], G122 is a cranberry bean [47] and both USPT-ANT-1 and Othello are pinto bean varieties [48, 49]. Within the differential cultivars Michelite is a navy bean [46], MDRK is an red kidney bean [50], Perry Marrow is a white marrow bean [51], Cornell 49,242 is a small black bean [50], Widusa is a snap bean [52], Kaboon is a white kidney bean [15], Mexico 222 a black bean [53], PI 207262 is a beige manteiga bean [54], TO is a dark beige seeded carioca bean [50], TU is a black bean [50], AB136 is a red bean [55] and G2333 is a maroon colored climbing bean [50]. The differential cultivars MDRK, Perry Marrow and Kaboon are Andean while the remaining are Mesoamerican [11, 52].

### DNA extraction

Before KASP assays could be conducted, DNA had to be extracted. For the manual procedure, DNA used was extracted from seed using a silica-based DNA extraction kit (DNeasy^®^ Plant Pro Kit, Catalogue number 69204). Before adding lysis buffers, seed was crushed manually and then ground into a powder using 4 mm stainless steel balls (SPEX^™^ sample prep 2150 grinding balls, Item number WZ-04500-19) in an automated tissue homogenizer (SPEX^™^ sample prep Geno/Grinder). A fine powder could be obtained with two 30-s cycles at 1500 rpm. The DNeasy^®^ Plant Pro extraction manual was followed from then on. DNA was extracted using this method from marker check lines and the differential anthracnose cultivars. DNA was also extracted manually from the differential cultivars using a magnetic bead-based plant DNA isolation kit (MagMAX^™^ Plant DNA Isolation Kit, Catalogue number: A32549) so that manual versus automated DNA yield could be quantified (Fig. 1).

For the automated procedure, DNA was extracted from seed using the same magnetic bead-based plant DNA isolation kit (MagMAX^™^ Plant DNA Isolation Kit, Catalogue number: A32549). Before adding lysis buffers, samples were ground as described above. Lysis steps were carried out manually before samples were put into the LHR for purification and elution. The LHR required the temperature block and magnetic module for DNA extraction. A protocol for DNA extraction using the MagMAX^™^ kit was developed on the Opentrons web protocol designer [56].

The concentration of eluted DNA was measured using a spectrophotometer (Thermo Fisher Nanodrop). DNA was then diluted down to the required concentration for KASP assays, 20–50 ng/μL, using ddH_2_O. Diluted DNA was run on a 1% agarose gel to confirm it had been successfully extracted and stored at -30℃ until KASP assays could be conducted.

### KASP assays

Two KASP assays were conducted by hand for each of the four markers tested as a comparison to the LHR. The assays involved an optimization experiment with positive and no template controls to optimize temperature settings, as well as a differential anthracnose bean cultivar assay. Three technical replicates were done for each sample. At least two no-template controls were used for each test; however, it was found that the master mix did fluoresce somewhat. We recommend running an additional well with ddH_2_O for added assurance. Primers were ordered from IDT [57], and the mastermix, sold as PACE 2.0 but functionally identical to KASP, was ordered from 3cr Bioscience [39]. The assays took place in a BioRad CFX connect PCR machine, and the allele calling function of CFX Maestro software was used to visualize the results. In some cases, human error caused poor results in one of the technical replicates. In those cases, only two technical replicates were used to make the call. The reaction volume was 10 µl consisting of 1 µl diluted DNA, 4 µl ddH_2_O, 5 µl PACE master mix, and 0.138 µl Assay mix containing both forward primers and the common reverse primer. Instructions for Pace 2.0 assay mix were followed.

The KASP protocol for the LHR was created and added on to the DNA extraction protocol. For this step, the thermocycler attachment was required. The protocols were developed using Opentrons online protocol building software. Both parts of the protocol for DNA extraction and KASP assaying in an LHR can be found at [58]. Thermocycling temperatures must be changed in the protocol to fit the markers being run.

One KASP assay was conducted with an LHR for each of the markers tested, the assay of the anthracnose differential bean cultivars (Table 1). Plate preparation and thermocycling took place inside the LHR. The reaction volume and components are the same as described above. Once the thermocycling was complete, endpoint fluorescence was read using the same BioRad CFX connect PCR, and results were again visualized using the CFX Maestro software.

### Supplementary Information

**Additional file 1: Figure S1.** Comparison of KASP results for the *Co-3*(A)*,Co-4*^*2*^ #1, (snpPV00070) (B) and *Co-4*^*2*^ #2, C) markers when done manually (left panels) versus by the LHR (right panels). The bean anthracnose differential cultivars were tested. Cultivars that are homozygous for resistance at the *Co-1* marker appear blue, while homozygous susceptible cultivars appear orange. Heterozygous samples are found between the two clusters. No template controls are either green or black. Two or three technical replicates are shown for the differential cultivars as well as check lines in the manual results.

**Additional file 2: Table S1.** KASP primer information for the four markers assayed.

## Data Availability

All timing data generated or analysed during this study are included in this published article and its supplementary information files. The LHR protocols generated during the current study are available in the Pulse Breeding Lab Github repository [58] at https://github.com/McGillHaricots/peas-andlove/tree/master/protocols.

## Abbreviations

KASP: Kompetitive allele-specific PCR
MAS: Marker assisted selection
LHR: Liquid handling robot
HEX: Hexachloro-fluorescein
FAM: Fluorescein amidites
qPCR: Quantitative PCR
MDRK: Michigan dark red kidney
GWAS: Genome wide association study
