# Supplementary figures and images for: Automating high-throughput screening for anthracnose resistance in common bean using allele specific PCR
Source: Plant Methods. 2023 Oct 3;19:102. doi: 10.1186/s13007-023-01071-5 (PMC10546687; doi:10.1186/s13007-023-01071-5)

A

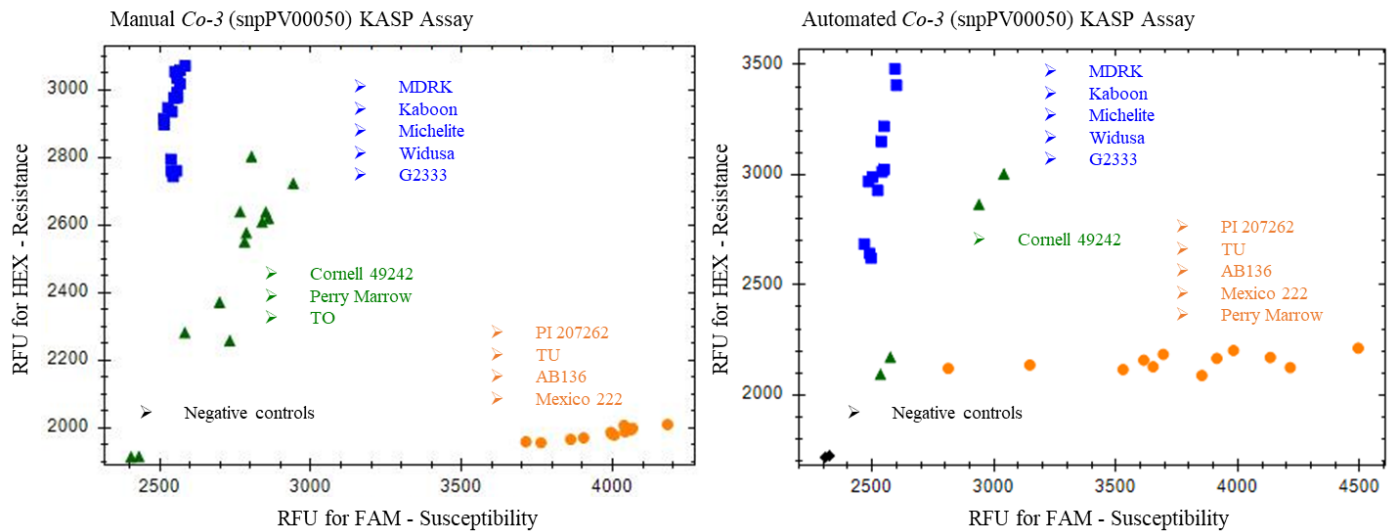

B

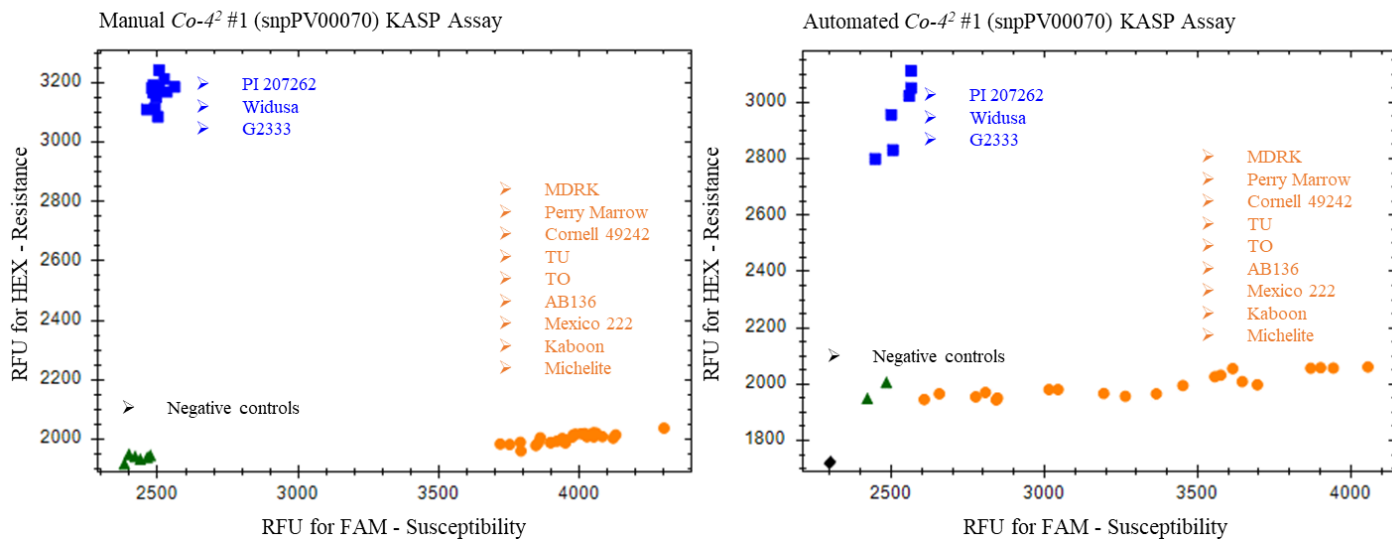

C

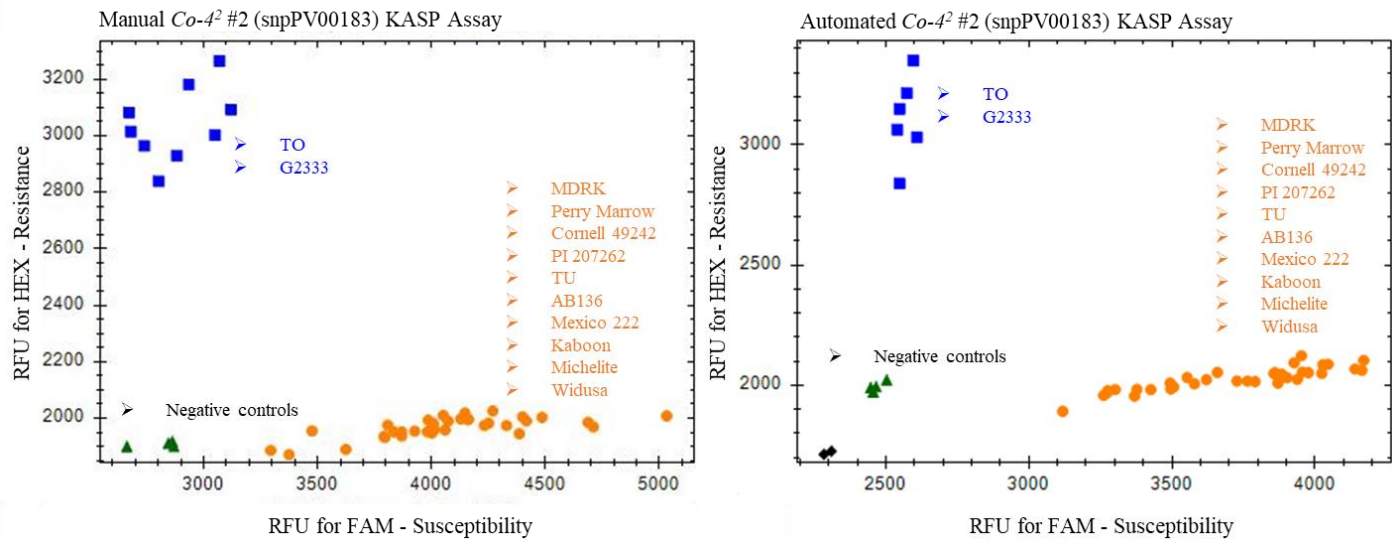

Supplement: Supplementary file 1 — Additional file 1: Figure S1. Comparison of KASP results for the Co-3(A),Co-42 #1, (snpPV00070) (B) and Co-42 #2, C) markers when done manually (left panels) versus by the LHR (right panels). The bean anthracnose differential cultivars were tested. Cultivars that are homozygous for resistance at the Co-1 marker appear blue, while homozygous susceptible cultivars appear orange. Heterozygous samples are found between the two clusters. No template controls are either green or black. Two or three technical replicates are shown for the differential cultivars as well as check lines in the manual results. [file 13007_2023_1071_MOESM1_ESM.pdf]
